# A Phase 2 clinical trial of PF-05212377 (SAM-760) in subjects with mild to moderate Alzheimer’s disease with existing neuropsychiatric symptoms on a stable daily dose of donepezil

**DOI:** 10.1186/s13195-018-0368-9

**Published:** 2018-04-05

**Authors:** Terence Fullerton, Brendon Binneman, William David, Marielle Delnomdedieu, James Kupiec, Peter Lockwood, Jessica Mancuso, Jeffrey Miceli, Joanne Bell

**Affiliations:** 1 Pfizer Global Product Development, 445 Eastern Point Rd., MS8260-2228, Groton, CT 06355 USA; 2 Pfizer Internal Medicine Research Unit, Cambridge, MA USA; 3 Pfizer Early Clinical Research, Cambridge, MA USA

**Keywords:** Alzheimer’s Disease, Serotonin, 5-HT

## Abstract

**Background:**

Symptomatic benefits have been reported for 5-HT_6_ receptor antagonists in Alzheimer’s disease (AD) trials. SAM-760 is a potent and selective 5-HT_6_ receptor antagonist that has demonstrated central 5-HT_6_ receptor saturation in humans at a dose of 30 mg.

**Methods:**

This was a randomized, double-blind, placebo-controlled, parallel-group, multicenter trial evaluating the efficacy and safety of SAM-760 30 mg once daily (QD) for 12 weeks in subjects with AD on a stable regimen of donepezil 5 to 10 mg QD. The study included an interim analysis with stopping rules for futility or efficacy after 180 subjects completed the week 12 visit. Up to 342 subjects with AD (Mini-Mental State Examination (MMSE) score 10–24) and neuropsychiatric symptoms (Neuropsychiatric Inventory (NPI) total score ≥ 10) were to be enrolled if the study continued after the interim analysis. After a 4-week, single-blind, placebo run-in period, subjects entered the 12-week double-blind period and were randomized to either SAM-760 or placebo. The primary and key secondary efficacy endpoints were the change from baseline in Alzheimer’s Disease Assessment Scale-cognitive subscale (ADAS-cog13) and NPI total scores. Mixed models for repeated measures were used to analyze the data.

**Results:**

At the interim analysis, when 186 subjects had been randomized and 163 had completed the week 12 visit, the study met futility criteria and was stopped. The mean week 12 treatment difference was 0.70 points (*P* = 0.43) for ADAS-cog13 and 2.19 points (*P* = 0.20) for NPI score, both of which were numerically in favor of placebo. Other secondary endpoints did not demonstrate any significant benefit for SAM-760. In total, 46.2% of SAM-760 subjects reported adverse events (AE) versus 44.7% for placebo, and there were 5 (5.5%) serious AEs in the SAM-760 group versus 3 (3.2%) for placebo. There were two deaths, one prior to randomization and one in the SAM-760 group (due to a traffic accident during washout of active treatment).

**Conclusions:**

SAM-760 was safe and well tolerated, but there was no benefit of SAM-760 on measures of cognition, neuropsychiatric symptoms, or daily function. Differences in trial design, study population, region, or pharmacological profile may explain differences in outcome compared with other 5-HT_6_ receptor antagonists.

**Trial registration:**

Clinicaltrials.gov, NCT01712074. Registered 19 October 2012.

**Electronic supplementary material:**

The online version of this article (10.1186/s13195-018-0368-9) contains supplementary material, which is available to authorized users.

## Background

Alzheimer’s disease (AD) is the most common form of dementia, affecting up to 5% of the population over the age of 65 years and 30% of those over the age of 80 years [1]. The pathology of AD is characterized by the presence of large numbers of amyloid plaques in the brain, neurons containing neurofibrillary tangles, and neurodegeneration [2]. Cognitive dysfunction, behavioral and mood disorders, and progressive memory loss represent the hallmark clinical features.

Acetylcholinesterase inhibitors are the mainstay of AD therapy, but their limited cognitive and behavioral effectiveness continues to underline an unmet medical need for additional, effective treatment options [3]. Even as newer disease-modifying treatments become available, there will still be a need for treatments that control or improve cognition and behavior.

The 5-HT_6_ receptor subtype of the serotonin (5-HT) receptor family has been implicated in learning, memory, and cognition [4–6], and may play a role in the etiopathology of neuropsychiatric symptoms in AD [7]. The potential for therapeutic use of 5-HT_6_ antagonists in the treatment of AD has been investigated in prior clinical trials that have demonstrated mixed results with respect to clinical efficacy. In clinical trials of SB-742457 (now known as intepirdine), administered as monotherapy to individuals with mild to moderate AD, there was no significant benefit of treatment versus placebo on measures of cognition or global impression of change, although some favorable trends were noted [8, 9]. In contrast, studies where 5-HT_6_ antagonists were added to existing stable regimens of cholinesterase inhibitors have produced more favorable results, with significant effects observed on measures of cognition and activities of daily living [10, 11]. The discrepant results between trials of 5-HT_6_ antagonist monotherapy and those assessing utility as an add-on therapy have been postulated to be due to the potentially synergistic mechanisms of potentiating acetylcholine release through 5-HT_6_ blockade while promoting synaptic neurotransmitter durability via cholinesterase inhibition [12]. Despite these inconsistent findings, the available data suggest that 5-HT_6_ antagonists may represent a viable additive treatment strategy to address symptoms of AD.

PF-05212377 (SAM-760) is a potent and selective antagonist of the human serotonin 5-HT_6_ receptor, with an average half-life that ranges from 27 to 34 h, that is a potential therapy for the symptomatic treatment of mild to moderate AD [13, 14]. A Phase 2a study was undertaken to evaluate the efficacy and safety of SAM-760 in subjects with mild to moderate AD with comorbid neuropsychiatric symptoms who were already on a stable daily dose of donepezil.

## Methods

### Study design

This was a randomized, 18-week (12-week treatment), double-blind, placebo-controlled, parallel-group, Phase 2a study in subjects with mild to moderate AD on a stable background daily dose of donepezil. The study was conducted at 37 centers: 25 in the USA, 1 in Canada, 3 in Chile, 5 in Germany, 1 in Spain, and 2 in the UK. The final study protocol, amendments, and informed consent documentation were reviewed and approved by the Institutional Review Boards or Ethics Committees at each participating center (listed in Additional file 1). The study was conducted in compliance with the declaration of Helsinki and in compliance with all International Conference on Harmonization Good Clinical Practice Guidelines. All participants (or their legal representative) provided written informed consent prior to screening.

**Fig. 1:**
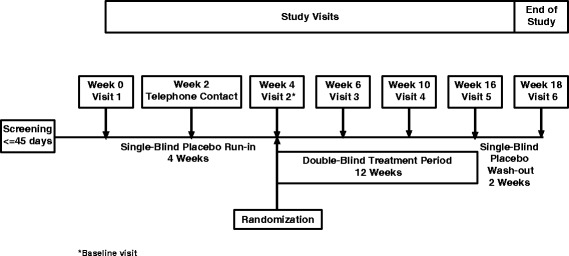
Study schematic

The primary objective was to evaluate the efficacy and safety of SAM-760 30 mg once daily (QD) in subjects with mild to moderate AD who had existing neuropsychiatric symptoms at both screening and baseline. The design included a 4-week, single-blind, placebo run-in period followed by a 12-week, double-blind treatment period in which subjects were randomized 1:1 to receive placebo or 30 mg QD SAM-760 (Fig. 1). The study utilized a central interactive voice response system (IVRS) to facilitate randomization. Randomization was stratified by country (USA, Canada, France, Germany, Spain, UK, Australia, Argentina, Chile) and disease status (based on the Mini-Mental State Examination (MMSE) score at visit 1: mild 20–24, moderate 10–19). Blocked randomization was used with a block size of 4 (2 on active, 2 on placebo). This was followed by a 2-week, single-blind placebo washout period. Overall medication compliance was calculated as the percentage of capsules taken of the total prescribed over the total treatment period.

The original analysis plan included two prespecified interim analyses for assessment of futility or early efficacy to be conducted after approximately 90 and 180 subjects had completed 12 weeks of treatment (week 16, visit 5) which represented approximately 31% and 62% of the targeted number of completers for the entire study, respectively. Prior to the first interim analysis, the study was modified to include only a single interim analysis for futility. This modification was made primarily for operational and business reasons, and also in consideration of recruitment projections which indicated that the number of subjects completing week 12 of double-blind therapy would likely approximate that originally targeted for the second interim analysis (*n* = 180). This change was reflected in a revised statistical analysis plan prior to the interim analysis.

An independent external Data Safety Monitoring Committee (DSMC) was constituted primarily to oversee matters of subject safety at the study level on an ongoing basis during the study. The DSMC reviewed aggregate and subject-level safety data in a masked fashion periodically during the study, with the ability to request unblinded data should any safety signals warrant the need to do so. The DSMC also reviewed the interim analysis efficacy data and was to make a recommendation to the sponsor Executive Steering Committee (ESC) who, under consultation with the DSMC, interpreted the interim analysis results according to the predefined stopping rules and who made the final decision to terminate the study. The ESC was firewalled from the study team who remained blinded to the study results until after study termination and final database release.

### Study population and sample size determination

The study population consisted of subjects with probable mild to moderate AD with existing neuropsychiatric symptoms on a stable dose of donepezil (5 or 10 mg daily for at least 4 months). Sample size calculations estimated that 342 subjects would need to be randomized to yield approximately 290 subjects completing 12 weeks of treatment (i.e., week 16, visit 5). This sample size was based on the number of subjects needed to result in an 80% chance of detecting a treatment group difference of at least 1.5 points in change from baseline in the 13-item version of the Alzheimer’s Disease Assessment Scale-cognitive subscale (ADAS-cog13) total score (based on a one-sided α = 10% two sample *t* test, assuming a common standard deviation of 6, and a drop-out rate of 15% of randomized subjects up to week 16 (visit 5)).

### Key inclusion/exclusion criteria

Key inclusion and exclusion criteria included (but were not limited to) the following. Subjects were required to be diagnosed with probable mild to moderate AD using clinical criteria of the Neurological and Communicative Disorders and Stroke – Alzheimer’s disease and Related Disorders (NINCDS-ADRDA) [15]. Subjects were also required to have an MMSE score of 10–24, inclusive, at screening and at entry into the single-blind run-in period at visit 1. The MMSE score could not deviate more than 3 points in either direction between the MMSE score at visit 1 and a repeat MMSE score obtained at visit 2 (baseline) prior to randomization. Additionally, all subjects were required to have been on a stable dose and regimen of donepezil 5 mg or 10 mg for at least 120 days prior to study visit 1, with no intent to change such during the study. Subjects were also required to have existing neuropsychiatric symptoms documented by a Neuropsychiatric Inventory (NPI) total score ≥ 10 at screening arising from at least two domains, and subjects also had to have a NPI total score ≥ 5 at visit 2 to be randomized. There was also a requirement for a qualified caregiver/informant to be available as a study partner and able to sign an informed consent along with the subject. Reasons for exclusion included major structural brain disease (e.g., ischemic infarcts, subdural hematoma, hemorrhage, hydrocephalus, brain tumors, multiple subcortical ischemic lesions, or a single lesion in a critical region (e.g., thalamus, hippocampus), or a degree of white matter disease sufficient to call into question the diagnosis of primary AD versus vascular dementia), severe acute or chronic medical or psychiatric conditions, or a laboratory abnormality that could have either increased the risk associated with study participation or interfered with the interpretation of the trial results. Use of memantine during the 30 days prior to visit 1 was also a reason for exclusion as was the use of acetylcholinesterase inhibitors, other than donepezil, within 120 days prior to visit 1 or planned use through or before the end of the study.

### Primary efficacy endpoint

The primary efficacy endpoint was the change from baseline (visit 2) to week 12 postrandomization (visit 5) in the total score for the 13-item version of the ADAS-cog13. This version of the ADAS-cog13 total score includes items for delayed recall and concentration/distractibility. Higher ADAS-cog13 total score represents a poorer outcome. A 2-point average change in ADAS-cog13 score versus placebo was considered clinically significant for the purposes of internal decision making for this proof of concept study.

### Secondary and exploratory efficacy endpoints

The secondary efficacy endpoint was the change from baseline to week 12 in NPI total score, with a higher score representing a poorer outcome. The following exploratory endpoints were also included: ADAS-cog11, MMSE, Alzheimer’s Disease Cooperative Study-Activities of Daily Living (ADCS-ADL), Clinician’s Global Impression of Improvement (CGI-I), Cornell Scale for Depression in Dementia (CSDD), and executive function (Category Fluency Test (CFT), Controlled Oral Word Association Test (COWAT)).

### Safety endpoints

Safety endpoints included the occurrence of adverse events (AEs) and serious AEs (SAEs). Physical examinations, electrocardiograms (ECGs), neurologic assessments, and laboratory analysis were also conducted. The Columbia Suicide Severity Rating Scale (CSSRS) was used to assess suicidal ideation and behavior.

### Pharmacokinetics

Plasma samples were collected in all subjects prior to dosing on the day of each visit for determination of SAM-760 and/or donepezil steady-state trough concentrations. The drug concentrations were determined using validated assays.

### Statistical methods

The primary analysis used a mixed model repeated measures (MMRM) method to assess change from baseline to week 12 postrandomization (visit 5). MMRM analysis assumes data missing at random which is reasonable given that, by design, the majority of discontinued subjects had study termination visits where endpoint data and reason for dropout were collected.

A Bayesian approach was used to conduct an interim analysis for futility. The predictive probability of a successful outcome for the primary endpoint (ADAS-cog13) at the end of the study (with 342 subjects) was calculated based on the interim analysis data. A successful outcome was predefined as *P*(ΔΔADAS-cog13 > 0) > 0.9 where ΔΔADAS-cog13 is the placebo-adjusted change from baseline to week 12 postrandomization in ADAS-cog13. Futility was to be declared if this predictive probability was less than 10%.

## Results

The trial was terminated after the interim analysis was conducted when 163 subjects had completed the week 12 posttreatment visit (visit 5); the analysis revealed the criteria for futility had been met. The Bayesian predictive probability for success on the ADAS-cog13 endpoint at study end was shown to be less than 1%, meaning that even if the trial had been continued to completion (*n* = 342) there was essentially no chance to demonstrate a benefit of SAM-760 over placebo on the primary endpoint. As the trial recruitment was paused for the interim analysis and no subjects were ongoing during the conduct of the interim analysis, the results below describe the final analysis of the trial.

**Fig. 2:**
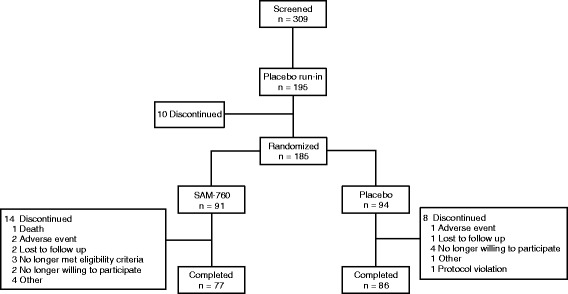
Subject disposition

In total, 309 subjects were screened and 195 entered the 4-week single-blind placebo run-in period (Fig. 2). Of the subjects who entered the run-in period, 92 and 94 subjects were randomized to the SAM-760 30 mg QD and placebo groups, respectively. Treatment groups were well-matched with regard to baseline demographics (Table 1). Of the 186 subjects randomized, 116 (62%) were from sites in the USA, while the remainder were from sites in Chile (*n* = 39), Canada (*n* = 16), Germany (*n* = 12), the UK (*n* = 2), and Spain (*n* = 1). One subject randomized to the SAM-760 group did not receive the study drug and was not included in the safety analysis. There were 14 and 8 discontinuations during the 12-week, double-blind treatment phase in the SAM-760 and placebo groups, respectively (Fig. 2).

**Table 1:** Baseline demographics

	SAM-760 30 mg QD (*n* = 92)	Placebo (*n* = 94)
Male, *n* (%)	46 (50.0)	39 (41.5)
Mean age, years (SD)	76.0 (8.0)	75.9 (7.5)
Mean weight, kg (SD)	72.4 (13.3)	70.8 (15.4)
Mean body mass index, kg/m^2^ (SD)	26.8 (4.6)	26.8 (4.3)
Mean height, cm (SD)	164.4 (9.6)	161.8 (11.1)
Mean duration since AD diagnosis, years	2.8	2.9
MMSE score mild (20–24), *n* (%)	53 (57.6)	47 (50)
MMSE score moderate (10–19), *n* (%)	39 (42.4)	47 (50)
Mean total MMSE score (SD)	19.7 (4.2)	19.6 (4.0)
Mean ADAS-cog13 score (SD)	34.9 (14.8)	33.2 (12.7)
Mean NPI score (SD)	21.1 (12.4)	21.8 (11.9)
Mean ADAS-cog11 score (SD)	25.7 (12.4)	24.1 (10.6)
Mean ADCS-ADL score (SD)	57.8 (13.7)	57.7 (15.7)
Mean CGI-I score (SD)	3.8 (0.8)	3.6 (0.6)
Mean CSDD score (SD)	5.9 (4.3)	5.3 (3.6)
Mean executive function composite score (SD)^a^	−0.1 (0.1)	0.1 (0.1)
ApoE4 genotype, *n* (%)		
E2/E4	2 (2.2)	2 (2.2)
E3/E4	37 (40.1)	41 (44.6)
E4/E4	7 (7.7)	4 (4.4)

^a^Composite of Category Fluency Test and Controlled Oral Word Association Test

*AD* Alzheimer’s disease, *ADAS-cog13/11* Alzheimer’s Disease Assessment Scale-cognitive subscale, *ADCS-ADL* Alzheimer’s Disease Cooperative Study-Activities of Daily Living, *CGI-I* Clinician’s Global Impression of Improvement, *CSDD* Cornell Scale for Depression in Dementia, *CFT* Category Fluency Test (executive function), *COWAT* Controlled Oral Word Association Test, *MMSE* Mini-Mental State Examination, *NPI* Neuropsychiatric Inventory, *QD* once daily, *SD* standard deviation

### Analysis of efficacy endpoints

**Fig. 3:**
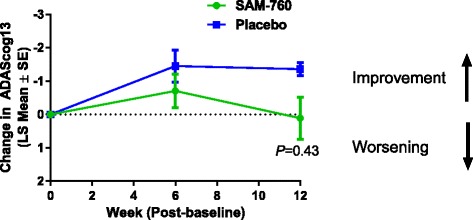
Least squares mean change from baseline in ADAS-cog13 score. *ADAS-cog13* Alzheimer’s Disease Assessment Scale-cognitive subscale, *LS* least squares, *SE* standard error

For the primary endpoint of change from baseline in ADAS-cog13 score, both SAM-760 and placebo treatment groups were near or improved from baseline at week 12. The least squares (LS) mean change from baseline in the ADAS-cog13 total score at week 16 was 0.11 in the SAM-760 group and −0.58 in the placebo group (a difference of 0.70, 80% confidence interval (CI) −0.42 to 1.81; *P* = 0.43). There was no evidence of a beneficial effect of SAM-760 over placebo at either postbaseline visit (week 6 or week 12). The ADAS-cog13 change from baseline score for SAM-760 was numerically inferior compared with placebo, although this difference was not statistically significant (Fig. 3 and Table 2).

**Table 2:** MMRM results for ADAS-cog13 at week 6 (visit 3) and week 12 (visit 5)

Visit	Treatment group	*n*	LS mean (SE)	80% CI	Difference (SE)	80% CI for difference	Two-sided *P* value
Week 6	SAM-760	84	−0.71 (0.50)	−1.36, −0.06	0.74 (0.70)	−0.16, 1.64	0.29
	Placebo	93	−1.45 (0.48)	−2.06, −0.83			
Week 12	SAM-760	78	0.11 (0.63)	−0.70, 0.92	0.70 (0.87)	−0.42, 1.82	0.43
	Placebo	86	−0.58 (0.60)	−1.36, 0.19			

*ADAS-cog13* Alzheimer’s Disease Assessment Scale-cognitive subscale, *CI* confidence interval, *LS* least squares, *MMRM* mixed model repeated measures, *SE* standard error

**Fig. 4:**
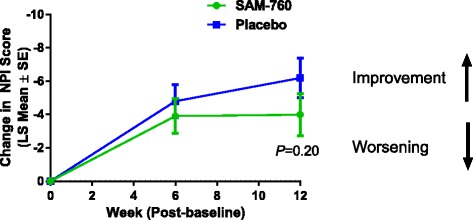
Least squares mean change from baseline in NPI score. *LS* least squares, *NPI* Neuropsychiatric Inventory, *SE* standard error

There was no evidence of a beneficial effect of SAM-760 over placebo at either postbaseline visit for any secondary or exploratory endpoint, including total NPI score (Fig. 4), with a LS mean change from baseline in the NPI total score at week 12 postbaseline of −3.99 and −6.18 in the SAM-760 and placebo groups, respectively (a difference of 2.19, 80% CI −0.01 to 4.40; *P* = 0.20). SAM-760 was numerically inferior to placebo for all endpoints except the CSDD; these differences were not statistically significant (Table 3).

**Table 3:** MMRM results for selected secondary and exploratory endpoints at week 12 (visit 5)

Endpoint	Treatment group	*n*	LS mean (SE)	80% CI	Difference (SE)	80% CI for difference	Two-sided *P* value
NPI total	SAM-760	78	−3.99 (1.24)	−5.59, −2.40	2.19 (1.71)	−0.01, 4.40	0.20
	Placebo	87	−6.18 (1.18)	−7.70, −4.67			
ADAS-cog11	SAM-760	78	0.26 (0.55)	−0.44, 0.97	0.65 (0.76)	−0.32, 1.62	0.39
	Placebo	87	−0.39 (0.52)	−1.05, 0.28			
ADCS-ADL	SAM-760	65	−0.69 (0.82)	−1.75, 0.37	0.82 (1.13)	−0.64, 2.28	0.47
	Placebo	72	−1.51 (0.78)	−2.51, −0.50			
CSDD	SAM-760	79	−1.41 (0.34)	−1.85, −0.97	− 0.59 (0.47)	−1.19, 0.02	0.22
	Placebo	86	−0.82 (0.32)	−1.24, −0.41			
CGI-I	SAM–760	80	3.83 (0.12)	3.68, 3.98	−0.03 (0.16)	−0.23, 0.18	0.87
	Placebo	86	3.86 (0.11)	3.71, 4.00			
MMSE	SAM-760	80	0.43 (0.29)	0.05, 0.80	−0.38 (0.40)	−0.90, 0.13	0.34
	Placebo	88	0.81 (0.28)	0.45, 1.16			

*ADAS-cog11* Alzheimer’s Disease Assessment Scale-cognitive subscale, *ADCS-ADL* Alzheimer’s Disease Cooperative Study-Activities of Daily Living, *CGI-I* Clinician’s Global Impression of Improvement, *CSDD* Cornell Scale for Depression in Dementia, *MMRM* mixed model repeated measures, *MMSE* Mini-Mental State Examination, *NPI* Neuropsychiatric Inventory

Additional post-hoc analyses were conducted to further evaluate the negative findings and to assess the potential for differential treatment effects across informative subgroups in the trial. Such analyses were solely for exploratory purposes in an attempt to further understand the futility outcome. These analyses suggested that baseline covariates (e.g., total NPI, disease status based on MMSE, age, sex, ApoE4 carrier status, country) did not appear to influence treatment response for ADAS-cog13 or NPI, although some subsets were too small to yield meaningful conclusions. There was no clear relationship between the magnitude of change in clinical status (as measured by MMSE, ADAS-cog11, or NPI) from screening to baseline and treatment response. Removal of statistical outliers (above the 75th percentile + 1.5 times interquartile range or below 25th percentile − 1.5 times interquartile range) did not change the overall interpretation. Due to the numerical imbalance in the number of discontinuations between the SAM-760 (*n* = 14) and placebo (*n* = 8) groups, a per-protocol analysis was performed on ADAS-cog and NPI to evaluate the sensitivity of the treatment response to this observation. No meaningful differences were observed between this completer analysis and the full analysis set for any of these endpoints. There was no apparent impact of concomitant psychotropic medication on the treatment effect for ADAS-cog13 or NPI.

### Pharmacokinetics

SAM-760 concentrations in the active treatment group and the donepezil concentrations in both the treatment and placebo groups were as expected. SAM-760 trough concentrations were generally consistent and stable across visits, indicating that steady-state exposure had been achieved, consistent with its half-life of 27 to 34 h and with previous pharmacokinetic data (on file) at the 30-mg daily dose. Mean SAM-760 plasma trough concentrations for the week 6, week 10, and week 16 visits ranged from 41.9 to 44.8 ng/ml. Variability in plasma concentrations at these visits, as quantified by the coefficient of variation (%CV), ranged from 38% to 42%.

For the SAM-760 and placebo treatment groups, mean donepezil plasma concentrations for the week 4, week 6, week 10, and week 16 visits ranged from 33.5 ng/ml to 41.4 ng/ml. Variability in plasma concentrations at these visits, as quantified by the %CV, ranged from 49% to 66%.

### Safety and tolerability

SAM-760 was generally safe and well tolerated, with a similar number of treatment-emergent adverse events (TEAEs) and SAEs in the SAM-760 and placebo groups, respectively (Table 4). There were two permanent discontinuations due to AEs in the SAM-760 group, one due to diarrhea and the other due to tinnitus, and one discontinuation in the placebo group due to pulmonary fibrosis. Overall, the most frequently reported AEs in either treatment group included diarrhea (8.8% SAM-760 versus 3.2% placebo), urinary tract infection (6.6% SAM-760 versus 4.3% placebo), and falls (3.3% SAM-760 versus 3.2% placebo). The rate of occurrence of SAEs during the double-blind treatment period was low: 2 (2.2%) subjects in the SAM-760 group (1 bradycardia and 1 acute cholecystitis) and 2 (2.1%) subjects in the placebo group (1 pneumonia and 1 accidental device ingestion). These SAEs were resolved and all were deemed to be unrelated to treatment. There were two deaths during the study: one subject died due to multi-organ failure at screening (prior to randomization) and one subject in the SAM-760 group died due to a road traffic accident 9 days after completing double-blind treatment. No treatment-related deaths or severe AEs occurred during the double-blind period of the study. Two subjects temporarily discontinued the study drug due to an AE: one in the SAM-760 group due to acute cholecystitis, and one subject in the placebo group due to pneumonia. Both discontinuations lasted 4 days, after which these AEs resolved and subjects continued to participate in the study.

**Table 4:** Summary of all-causality treatment-emergent AEs

	Placebo run-in (*n* = 195)	SAM-760 (*n* = 91)^a^	Placebo (*n* = 94)	Placebo washout (*n* = 164)
Number of AEs	39	53	62	22
Number of subjects (%)				
With AEs	25 (12.8)	38 (41.8)	39 (41.5)	19 (11.6)
With serious AEs	1 (0.5)	2 (2.2)	2 (2.1)	4 (2.4)
With severe AEs	2 (1.0)	0	0	2 (1.2)
Discontinued due to AEs	2 (1.0)	2 (2.2)	0	1 (0.6)
Dose reduced or temporary discontinuation due to AEs	1 (0.5)	2 (2.2)	1 (1.1)	0

^a^One subject was randomized but not treated; therefore *n* = 91 not *n* = 92

*AE* adverse event

There were no discernible differences between treatment groups with respect to hematology or clinical chemistry, physical examinations, vital signs, ECGs, or suicidal ideation and behavior as determined by CSSRS.

## Discussion

In this study, administration of SAM-760 30 mg QD for 12 weeks to subjects with mild to moderate AD with existing neuropsychiatric symptoms and receiving a stable daily dose of donepezil was generally safe and well tolerated. Despite a human positron emission tomography (PET) study indicating saturation of the 5-HT_6_ receptor at the 30-mg QD dose [13], there was no significant difference compared with placebo for the primary endpoint (ADAS-cog13). Similarly, there was no significant difference for the key secondary endpoint of NPI total score, or for any of the exploratory efficacy parameters (MMSE, ADAS-cog11, CDSS, CGI, and ADCS-ADL). In fact, in all cases except the CSDD, numerical differences were in favor of placebo, indicating no trends for any treatment benefits in this study. The predictive probability of a successful outcome (i.e., *P*(ΔΔADAS-cog13 > 0) > 0.9) in favor of SAM-760 had the study been continued to completion (with 342 subjects) was less than 1%. This prediction, together with the consistency of inferences about treatment effect reported above and for the per-protocol analysis, strongly suggest that the inference regarding treatment effectiveness was not likely impacted by suboptimal power. Results of the per-protocol analysis on ADAS-cog and NPI also did not change the inferences regarding treatment effect. Plasma concentrations of SAM-760 were consistent with expectations and reached steady-state exposure after 2 weeks. Similarly, plasma donepezil concentrations were consistent with previous reports in the literature [16]. These findings support the notion that subjects were generally compliant with the dosing schedule of study drug and donepezil during the trial.

As discussed, the study was terminated for futility immediately after the interim analysis due to the extremely low likelihood that continuation of the study would have resulted in a measurable separation between treatment groups in favor of SAM-760 for the primary efficacy endpoint of improvement in ADAS-cog13 score. There were no safety concerns identified by the independent external DSMC, and none were obvious by review of the final safety results of this study.

An explanation as to why the results of this study differ from other 5-HT_6_ receptor antagonist Phase 2 studies could not be identified, but may be due to differences in trial design, or differences in the characteristics of the study populations and the regions in which the studies were conducted. To explore the potential for differentiation of 5-HT_6_ receptor antagonists from other symptomatic agents, we purposely sought to explore the possible beneficial effects of SAM-760 on measures of neuropsychiatric symptoms, and therefore enriched the trial with subjects with existing symptoms at baseline. It is possible that these subjects were more refractory to any procognitive effects of SAM-760, thus contributing to the negative results. Previous published studies of other 5-HT_6_ receptor antagonists did not select participants for the presence of neuropsychiatric symptoms and this therefore could explain the discrepant findings between the previous results and those reported here.

Another potential explanation could involve the prohibited use of memantine in this study. The majority of sites (25/37) participating in this trial were based in the USA where memantine is frequently prescribed in conjunction with cholinesterase inhibitors throughout the AD severity spectrum. Indeed, this restriction proved to be a significant barrier to recruitment to this study in the USA. It is therefore possible that US subjects (62% of randomized subjects) who were not receiving memantine as standard of care (and thus eligible for participation in this trial) could represent an atypical population and might potentially be more resistant to pharmacologic treatment with other therapies. However, since neither details of potential previous memantine use nor reasons for its nonutilization in subjects enrolled in this trial were captured, this point remains speculative. The published positive trial of idalopirdine in moderate AD did not report exclusion of concomitant memantine use as a criterion for eligibility. However, this study was conducted entirely outside of the USA, where memantine use is not uniformly considered standard of care, and therefore it not clear how allowance of memantine might have influenced the outcome [11]. The authors did not report the extent of memantine use by trial participants. In contrast, the successful intepirdine Phase 2 trial did not permit coadministration of memantine. This study was conducted, in part, in the USA; however, the publication did not indicate the proportion of subjects enrolled from the USA. The intepirdine trial was significantly larger and included a larger number of countries beyond the USA than the SAM-760 trial reported here, in which approximately 62% of randomized subjects were from the USA.

It is also possible that inherent differences between different 5-HT_6_ antagonist molecules in terms of their specific mode of action or off-target effects could have contributed to the differences in observed efficacy profiles across studies. For example, a previous PET receptor occupancy study suggested that the 5-HT_6_ antagonist intepirdine may also bind to the 5-HT_2a_ receptor, whereas a PET study of SAM-760 using a more specific 5-HT_2a_ ligand showed no evidence of 5-HT_2a_ binding activity. However, no direct comparison can be made because the two compounds have not been studied using the same 5-HT_2a_ ligand [13, 14, 17].

It has been reported that multiple Phase 3 studies of idalopirdine conducted in patients with mild to moderate AD failed to meet their primary endpoints of improvement in ADAS-cog score [18, 19]. More recently, the results of the intepirdine Phase 3 trial were reported and indicated that the study also failed to meet its coprimary endpoints, suggesting that the 5-HT_6_ mechanism may not be a viable strategy for treatment of the cognitive and functional impairments in AD [20].

One can only speculate on why the results of the idalopirdine and intepirdine Phase 3 trials were different from the Phase 2 results. There were distinct differences in the designs and, for idalopirdine, the dose regimens employed in these Phase 3 trials compared with the positive Phase 2 studies. These differences could explain the discrepant findings [11]. It is also possible that the true magnitude of any beneficial cognitive effect of a 5-HT_6_ receptor antagonist is exceedingly small, increasing the potential for a false-negative result with even the relatively large sample sizes employed in the three trials in the idalopirdine and intepirdine Phase 3 program. Indeed, for intepirdine, there was a small, nonsignificant trend reported for mean ADAS-cog change from baseline of 0.36 points (*P* = 0.22) and a statistically significant result on the mean Clinician Interview-Based Impression of Change plus Caregiver input (CIBIC+) secondary endpoint of 0.12 points (*P* = 0.02). Such changes are of no clinical relevance, and the results reflect the large sample size employed in the Phase 3 trial [20].

It is also important to note that selection of only the positive Phase 2 clinical trials across previous results for different 5-HT_6_ antagonists in the decision to proceed to pivotal studies, and discounting the negative monotherapy trials that were reported for intepirdine, poses a danger of both bias and inflation of the assumed treatment effect size for such agents. Such overestimation of the presumed treatment effect based on a single random high Phase 2 study result is an example of the regression to the mean phenomenon that is well known in drug development [21]. The fact that most secondary endpoints in both the positive Phase 2 idalopirdine and intepirdine trials were not positive, despite some being potentially more sensitive measures of cognition than the ADAS-cog, could support the notion of a very small overall true magnitude of treatment benefit for 5-HT_6_ receptor antagonists, as discussed above. This point was raised by Schneider in the editorial accompanying the idalopirdine LADDER results [22].

Despite the early promise that antagonism of the 5-HT_6_ receptor would be a useful treatment strategy for AD, the failure of SAM-760 to demonstrate efficacy in this study, along with the recently reported failures of idalopirdine and intepirdine to demonstrate efficacy in multiple larger scale Phase 3 clinical trials, casts significant doubt on the viability of 5-HT_6_ as a target for AD treatment. While the precise reasons for the negative outcomes of this Phase 2 trial and the Phase 3 trials with idalopirdine and intepirdine are unknown, the most parsimonious explanation is that the mechanism does not demonstrate sufficient potency as a modulator of cholinergic neurotransmission, either alone or when administered in conjunction with an acetylcholinesterase inhibitor, to yield demonstrable clinical benefit in patients with AD.

Intepirdine remains in clinical development for dementia with Lewy bodies (DLB), a disorder with a greater degree of cholinergic dysfunction that could be more responsive to subtle effects of this mechanism, and with a 70-mg top clinical dose, which could lead to a greater degree of 5-HT_2A_ blockade and resulting benefits on other aspects of DLB beyond cognition. Time will tell whether this alternative indication will prove to be more tractable for this mechanistic approach.

## Conclusions

While our study failed to demonstrate a treatment effect of 5-HT_6_ antagonism with SAM-760, this work contributes to the growing body of literature with this approach. When interpreted in light of the recent Phase 3 results of other 5-HT_6_ antagonists, it would appear that this mechanism has been sufficiently tested to conclude a clear lack of utility in AD.

## Additional file

Additional file 1: List of Institutional Review Boards/Independent Ethics Committees. (DOCX 17 kb)

## Abbreviations

5-HT: Serotonin
AD: Alzheimer’s disease
ADAS-cog: Alzheimer’s Disease Assessment Scale-cognitive subscale
ADCS-ADL: Alzheimer’s Disease Cooperative Study-Activities of Daily Living
AE: Adverse event
CFT: Category Fluency Test
CGI-I: Clinician’s Global Impression of Improvement
CI: Confidence interval
COWAT: Controlled Oral Word Association Test
CSDD: Cornell Scale for Depression in Dementia
CSSRS: Columbia Suicide Severity Rating Scale
CV: Coefficient of variation
DLB: Dementia with Lewy bodies
DSMC: Data Safety Monitoring Committee
ECG: Electrocardiogram
ESC: Executive Steering Committee
LS: Least squares
MMRM: Mixed Model Repeated Measures
MMSE: Mini-Mental State Examination
NINCDS-ADRDA: Neurological and Communicative Disorders and Stroke – Alzheimer’s disease and Related Disorders
NPI: Neuropsychiatric Inventory
PET: Positron emission tomography
QD: Once daily
SAE: Serious adverse event
TEAE: Treatment-emergent adverse event
