# Supplementary material for: A Phase 2 clinical trial of PF-05212377 (SAM-760) in subjects with mild to moderate Alzheimer’s disease with existing neuropsychiatric symptoms on a stable daily dose of donepezil
Source: Alzheimers Res Ther. 2018 Apr 5;10:38. doi: 10.1186/s13195-018-0368-9 (PMC5887246; doi:10.1186/s13195-018-0368-9)
Supplement: Supplementary file 1 — List of Institutional Review Boards/Independent Ethics Committees. (DOCX 17 kb) [file 13195_2018_368_MOESM1_ESM.docx]

**Additional file 1**

**List of Institutional Review Boards/Independent Ethics Committees**

**Australia**

Bellberry Human Research Ethics Committee

129 Glen Osmond Road

Eastwood, SA 5063

AUSTRALIA

**Canada**

Queen's University Health Sciences and affiliated Teaching Hospitals Research Ethics Board

Fleming Hall/Jemmett Wing, 3rd floor

78 Fifth Field Company Lane

Kingston, ON K7L 3N6

CANADA

Conjoint Health Research Ethics Board

2500 University Drive NW, Mackimmie Library Tower (MLT 300) - 3rd Floor

Calgary, AB T2N 1N4

CANADA

University of Saskatchewan BioMedical Research Ethics Board (Bio-REB)

1607 - 110 Gymnasium Place, Research Ethics Office University of Saskatchewan NRC - Plant Biotechnology Research Institute

Saskatoon, SK S7N 4J8

CANADA

IRB Services (Institutional Review Board Services)

372 Hollandview Trail, Suite 300

Aurora, ON L4G 0A5

CANADA

Research Ethics, Human Research Protections Program, Sunnybrook Health Sciences Centre

2075 Bayview Avenue, Room C819

Toronto, ON M4N 3M5

CANADA

**Chile**

Servicio de Salud Metropolitano Oriente Comite de Etica Cientifico

Av. El salvador 364, Providencia

Santiago, METROPOLITANA 7500922

CHILE

**France**

CPP du SUD-OUEST et OUTRE-MER IV

Centre Hospitalier Esquirol Cabanis Haut

15 rue du Docteur Marcland

Limoges Cedex, 87025

FRANCE

**Germany**

Ethik-Kommission der Bayerischen Landesaerztekammer

Muehlbaurstrasse 16

Muenchen, 81677

GERMANY

**Spain**

CEIC Area 11 - Hospital 12 de Octubre

Avda. de Cordoba, s/n

Madrid, MADRID 28041

SPAIN

CEIC Hospital General Universitario de Elche

C/ Camino de la almazara, 11

Elche, 03203

SPAIN

Secretaría del Comité Etico de Investigación Clínica

Hospital General Yague, Avda. del Cid 96, Unidad de Investigación

Burgos, 09005

SPAIN

CEIC Área 5 - Hospital Universitario La Paz

Paseo de la Castellana, 261 Planta 8ª del Hosp. Gral.

Madrid, 28046

SPAIN

CEIC Hospital Mútua de Terrassa

Plaça Dr. Robert, 5

Terrassa, 08221

SPAIN

CEIC-Euskadi

Departamento de Sanidad del Gobierno Vasco.

Dirección de Farmacia.C/ Donostia-San Sebastián, nº 1., Comité Ético de Investigación Clínica de la Comunidad Autónoma del País Vasco.

CEIC-E

Victoria, 01010

SPAIN

CEIC Fundació de Gestió Sanitària Hospital de la Santa Creu i Sant Pau

Servicio de Farmacología Clínica - Pabellón 19

Av. Sant Antoni M. Claret, 167

Barcelona, 08025

SPAIN

CEIC Área 2 -Hospital Universitario de La Princesa

C/ Diego de León, 62

Madrid, 28006

SPAIN

**United Kingdom**

NRES Committee East Midlands - Nottingham 2

The Old Chapel, Royal Standard Place

Nottingham, NG1 6FS

UNITED KINGDOM

**United States**

Schulman Associates Institutional Review Board, Inc

4445 Lake Forest Dr, Ste 300

Cincinnati, OH 45242

UNITED STATES

Western Institutional Review Board

3535 Seventh Ave, Sw

Olympia, WA 98502

UNITED STATES

Human Investigative Committee

55 College St, Yale University School of Medicine

New Haven, CT 06510

UNITED STATES

Partners Human Research Committee

116 Huntington Ave, Ste 1002

Boston, MA 02116

UNITED STATES

Roper St. Francis HealthCare IRB

316 Calhoun St, 5th Fl

Charleston, SC 29401

UNITED STATES

Dean Institutional Review Board

2711 Allen Blvd, Ste 300

Middleton, WI 53562

UNITED STATES

Medical College of Wisconsin Froedtert Hospital

8701 Watertown Plank Rd, Institutional Review Board Human Research Review Committee

Milwaukee, WI 53226

UNITED STATES

Berkshire Medical Center Institutional Review Board

725 N St

Pittsfield, MA 01201

UNITED STATES

Johns Hopkins Medicine Office of Human Subjects Research Institutional Review Boards

1620 McElderry St, Reed Hall B130

Baltimore, MD 21205

UNITED STATES

Michigan State University IRB

Human Research Protection Program, Rm 207 Olds Hall, 408 W Circle Dr

East Lansing, MI 48824

UNITED STATES
